# A Novel Mode of Application of Hemp

**Published:** 1872-12

**Authors:** 


### A NOVEL MODE OF APPLICATION OF HEMP.

The Dublin Obstetrical Society, at one of its recent meetings, devoted the greater part of an evening to the discussion of the use of hemp ligature as a saw, for the removal of intra-uterine and vaginal tumors, and for decapitation in cases of transverse fœtal position in labor. Dr. McClintock spoke highly of this method in extirpating polypi, and reported a case.

A married lady came to him, reporting severe and repeated vaginal hemorrhage. A large polypus was discovered in the vagina, its size that of a turkey's egg, and its neck an inch in diameter. The tumor was drawn well down towards the vulva, and a loop of fishing line, introduced through Gooch's double canula, was cast around the pedicle, and in thirty-five seconds, by a sawing movement, the section through the neck of the growth was completed. The operation was facile, expeditious, dry, painless. The ligature was used through the canula, with a view to protect the vagina from injury by friction.

Dr. Kidd exhibited the body of a fœtus decapitated in the process of delivery by the method under discussion. The child was presenting transversely, and was dead. The cord was passed around the neck of the fœtus by means of a catheter, and the section was completed in a minute and a half. The whole operation of delivery was accomplished in twenty minutes. Dr. Kidd remarked, that if the practitioner had a cephalotribe beside him he need not fear the risk of the head remaining in the uterus.—*Dublin Medical Journal and Louisville & Richmond Med. Journal.*

